# Changes in kidney function among men having sex with men starting on demand tenofovir disoproxil fumarate – emtricitabine for HIV pre‐exposure prophylaxis

**DOI:** 10.1002/jia2.25420

**Published:** 2020-02-22

**Authors:** Geoffroy Liegeon, Guillemette Antoni, Gilles Pialoux, Catherine Capitant, Laurent Cotte, Isabelle Charreau, Cécile Tremblay, Eric Cua, Eric Senneville, François Raffi, Laurence Meyer, Jean‐Michel Molina, for the ANRS‐IPERGAY study group, L Meyer, C Capitant, I Charreau, E Netzer, N Leturque, J Binesse, V Foubert, M Saouzanet, F Euphrasie, D Carette, B Guillon, Y Saïdi, J P Aboulker, B Spire, M Suzan, G Cattin, B Demoulin, L Sagaon‐Teyssier, N Lorente, V Doré, E Choucair, S Le Mestre, A Mennecier, N Etien, M C Simon, A Diallo, S Gibowski, J F Delfraissy, D Thompson, J Sas, J Pankovitch, M Klein, A Anis, Jean‐Michel Molina, Mark A Wainberg, Benoit Trottier, Cécile Tremblay, Jean‐Guy Baril, Gilles Pialoux, Laurent Cotte, Antoine Chéret, Armelle Pasquet, Eric Cua, Nolwenn Hall, Willy Rozenbaum, Christian Chidiac, Constance Delaugerre, Nathalie Bajos, Julie Timsit, Gilles Peytavin, Julien Fonsart, Isabelle Durand‐Zaleski, Laurence Meyer, Jean‐Pierre Aboulker, Bruno Spire, Marie Suzan‐Monti, Gabriel Girard, Daniela Rojas Castro, Marie Préau, Michel Morin, David Thompson, Catherine Capitant, Anaïs Mennecier, Elias Choucair, Véronique Doré, Marie‐Christine Simon, Isabelle Charreau, Joanne Otis, France Lert, Alpha Diallo, Séverine Gibowski, Cecile Rabian

**Affiliations:** ^1^ Hôpital Saint‐Louis Assistance Publique Hôpitaux de Paris Paris France; ^2^ INSERM Villejuif France; ^3^ Hôpital Tenon Paris France; ^4^ Hôpital de la Croix Rousse Hospices Civils de Lyon Lyon France; ^5^ Centre Hospitalier de l'Université de Montréal Montréal Canada; ^6^ Hôpital de l'Archet Nice France; ^7^ Hôpital G. Dron Centre Hospitalier Universitaire de Tourcoing Tourcoing France; ^8^ INSERM UIC 143 Nantes University Nantes France; ^9^ Services des Maladies infectieuses Centre hospitalier universitaire de l'Hôtel‐Dieu Nantes France; ^10^ Université Paris Sud Paris Saclay France; ^11^ Université de Paris Diderot Paris 7 Sorbonne Paris Cité Paris France; ^12^ INSERM UMR 944 Paris France

**Keywords:** PrEP, on‐demand, intermittent, kidney, eGFR, tenofovir, HIV

## Abstract

**Introduction:**

Daily pre‐exposure prophylaxis (PrEP) with tenofovir disoproxil fumarate/emtricitabine (TDF/FTC) is associated with a small but statistically significant decrease in estimated glomerular filtration rate (eGFR). We assessed the renal safety of on‐demand PrEP with TDF/FTC in HIV‐1 uninfected men.

**Methods:**

We used data from the randomized double‐blind placebo‐controlled ANRS‐IPERGAY trial and its open‐label extension conducted between February 2012 and June 2016 among HIV‐uninfected MSM starting on‐demand PrEP. Using linear mixed model, we evaluated the mean eGFR decline from baseline over time and determined risks factors associated with eGFR decline during the study.

**Results:**

During the blind phase, with a median follow‐up of 9.4 months, the mean decline slope of eGFR from baseline was −0.88 and −1.53 mL/min/1.73 m^2^ per year in the placebo (n = 201) and the TDF/FTC group (n = 198) respectively, with a slope difference of 0.65 mL/min/1.73 m^2^ per year (*p* = 0.27). Including both phases, 389 participants started on‐demand TDF/FTC with a median follow‐up of 19.2 months and a mean decline of eGFR from baseline of −1.14 mL/min/1.73 m^2^ per year (*p* < 0.001). The slope of eGFR reduction was not significantly different in participants with baseline eGFR ≤ 90 mL/min/1.73 m^2^ (*p* = 0.44), age >40 years (*p* = 0.24) or hypertension (*p* = 0.21). There was a dose‐response relationship between recent tenofovir exposure and lower eGFR when considering the number of pills taken in the two months prior the visit (eGFR difference of −0.88 mL/min/1.73 m^2^ between >15 pills/month vs. ≤15 pills/month, *p* < 0.01) or plasma tenofovir concentrations at the visit (eGFR difference compared to ≤2 ng/mL: >2 to ≤10ng/mL: −0.98 mL/min/1.73 m^2^, >10 to ≤40ng/mL: −1.28 mL/min/1.73 m^2^, >40 ng/mL: −1.82 mL/min/1.73 m^2^, *p* < 0.001). Three participants discontinued TDF/FTC for eGFR < 60 mL/min/1.73 m^2^ during the OLE phase. No case of Fanconi syndrome was reported.

**Conclusions:**

The renal safety of on‐demand PrEP with TDF/FTC was good. The overall reduction and intermittent exposure to TDF/FTC may explain this good renal safety.

## 1 Introduction

Pre‐exposure prophylaxis (PrEP) with tenofovir disoproxil fumarate (TDF) – emtricitabine (FTC) raises a lot of expectations to hamper HIV epidemic due to its high effectiveness to prevent HIV acquisition in individuals at high risk of infection 1, 2, 3, 4, 5, 6, 7. However, as already reported in HIV‐infected patients 8, 9, both PrEP clinical trials and cohorts have shown that the daily use of TDF/FTC in men who have sex with men (MSM), intravenous drug users or serodiscordant couples was associated with a small but statistically significant decrease in estimated glomerular filtration rate (eGFR) and/or creatinine clearance 10, 11, 12, 13, 14, 15, especially in users with a baseline eGFR < 90 mL/min/1.73 m^2^, an age >40 years or with higher tenofovir exposure 13, 14, 15. With the global rollout of PrEP programmes, this concern merits close consideration and highlights the need to find new strategies to improve the renal safety of PrEP.

In 2015, the ANRS‐IPERGAY placebo‐controlled trial have shown that a “on‐demand” PrEP regimen was associated with a relative reduction in HIV infection of 86% among MSM 6. Based on evidence from this trial, on‐demand PrEP is now recommended by the European AIDS Clinical Society (EACS) 16 and the International Antiviral Society‐USA Panel 17 among MSM. In the ANRS‐IPERGAY study, on‐demand PrEP allowed to reduce by half the cumulative exposure to TDF/FTC compared to daily PrEP, with a median number of 15 pills taken per month. In addition, intermittent PrEP exposure may also allow reversibility of eGFR reduction during periods off PrEP as previously reported among HIV‐infected patients and PrEP users 18, 19. However, it is uncertain whether the overall reduction and intermittent exposure to TDF/FTC allowed by on‐demand PrEP could improve the renal safety of PrEP.

We aimed in this study to assess the renal safety of on‐demand PrEP with TDF/FTC in MSM by taking advantage of data collected prospectively during the ANRS‐IPERGAY trial and its subsequent open‐label extension 6, 20.

## 2 Methods

### 2.1 Study design and participants

The ANRS‐IPERGAY study was a double‐blind, randomized placebo‐controlled trial of on‐demand TDF/FTC for PrEP (https://ClinicalTrials.gov number, NCT01473472) conducted in six study sites in France and one in Canada 6. Participants eligible for the study were HIV‐negative MSM or transgender women, aged 18 years or older, who had unprotected anal sex with at least two different partners over the previous six months. Participants with a creatinine clearance below 60 mL/min estimated by Cockcroft and Gault equation and glycosuria or proteinuria of more than 1 + in urine dipsticks were not included in this study. From February 2012, 400 participants were randomly assigned to take a fixed dose combination of TDF/FTC (300mg TDF with 200 mg of FTC) or a matching placebo before and after sexual activity. For each sexual intercourse, participants took a loading dose of two pills with food two twenty‐four hours before sex, a third pill 24 hours after the first drug intake, and a fourth pill 24 hours later. In case of multiple consecutive sexual intercourses, participants took a pill per day until the last sexual intercourse followed by the two postexposure prophylaxis pills. In October 2014, after a median follow‐up of 9.3 months, the DSMB recommended that the placebo group be discontinued and that all the study participants be offered on‐demand PrEP with TDF/FTC. Between November 2014 and January 2015, 361 participants were enrolled in the OLE study 20. Participants were followed up until 30 June 2016, a date at which it was likely that TDF/FTC‐based PrEP would be approved in France.

### 2.2 Procedures

We scheduled study visits for each participants at enrolment, four weeks and eight weeks later, and every eight weeks thereafter. Serum creatinine and urine dipstick testing for proteinuria and glycosuria was measured at each visit. Creatinine clearance was estimated by Cockcroft and Gault equation to determine participant eligibility and monitor renal function. In this study, we used the Chronic Kidney Disease Epidemiology Collaboration (CKD‐EPI) equation to calculate eGFR which provides a more accurate estimate of GFR 21. To assess drug adherence, participants returned their study‐drug bottles at each visit to perform a pill count of unused medication. We also measured tenofovir levels in plasma in all participants at each visit on stored plasma using a validated liquid chromatography‐tandem mass spectrometry method, with a limit of quantification of 1 ng/mL for tenofovir 22. Adverse events were recorded at each visit and renal toxicity was graded according to the scale of the severity of adverse events in adults used by the France Recherche Nord et Sud Sida‐HIV et Hépatites (National Agency of Research on AIDS and Viral Hepatitis (ANRS)) 23.

### 2.3 Statistical analysis

Two samples for analyses were defined: the “blind phase sample” including all the 201 placebo and 199 TDF/FTC, and the “on TDF/FTC sample” including all data from blind and OLE phases of all the participants who initiated on‐demand TDF/FTC based PrEP during the study (199 participants from the blind phase, 161 participants from the placebo group who continued the study in the OLE phase and 29 new participants who started TDF/FTC during the OLE phase). The follow‐up of the “on TDF/FTC sample” started the day of the first prescription of on‐demand PrEP with TDF/FTC and ended at the last visit. Baseline characteristics were compared by Wilcoxon rank‐sum test for continuous variables and chi‐squared or Fisher's exact tests for qualitative variables. The evolution of eGFR over time was modelled by a mixed model with random intercept and slope. In the blind phase sample analysis, the arm, the time and the interaction between arm and time were included in the model. The slope difference between the two arms was assessed through testing an interaction term between arm and time. In the “on TDF/FTC” sample analysis, the association between baseline risk factors and eGFR decline was studied by testing an interaction term between risk factors and time. Covariates associated either with baseline eGFR or with eGFR decline at a *p* < 0.30 were included in the multivariate model. The mixed model also allowed us to study the association between the recent exposure to TDF/FTC, that is, just prior to the visit, and eGFR at the time of the visit, using pill intake (≤ or >15 pills/month in the last two months) or tenofovir plasma concentration at the time of visit, which reflects the exposure to TDF/FTC in the prior week. Finally, in order to compare eGFR decline according to TDF/FTC use in the study, two profiles of PrEP users were defined according to the number of pills taken throughout follow‐up: “low users” who have taken 15 pills or less per month at ≥75% of visits and other participants. The analysis of risk factors of eGFR decline was carried out by modelling a single slope. The Kaplan‐Meier method was used to assess the probability of eGFR falling to <70 mL/min/1.73 m^2^ during the study and a Log rank test was used to compare the two arms. This threshold has already been used in previous studies conducted in PrEP users 13, 14 and alerts the clinician on the need to strengthen renal monitoring or to discontinue TDF/FTC prophylaxis. The Kaplan‐Meier method was also used to evaluate the probability of having a recurrence of eGFR < 70 after a first event. All *p* values and confidence intervals were two‐sided. All analyses were conducted with R version 3.5.2.

### 2.4 Ethical consideration

The ANRS‐IPERGAY trial protocol and the amendment to implement the open‐label extension study were approved by public health authorities and by ethics committees in France (Comité de Protection des Personnes, Paris, Ile de France IV) and Canada (Comité d'éthique de la recherche, Montreal, QC). All participants enrolled in the parent study provided a written consent authorizing the use of their clinical and laboratory data for research purposes and publication.

## 3 Results

### 3.1 Characteristics of IPERGAY participants at baseline

From February 2012 through October 2014, 201 participants were enrolled in the placebo group and 199 in the TDF/FTC group. The baseline characteristics were similar in the two treatment groups (Table 1). The median plasma creatinine level was 81 µmoL/L (interquartile range IQR 75 to 88) in the TDF/FTC arm and 82 µmoL/L (73 to 87) in the placebo arm (*p = *0.52) with median eGFR values of 106 (97 to 115) and 108 mL/min/1.73 m^2^ (96 to 115) respectively (*p* = 0.44). The median follow‐up of the blind phase was 9.4 months (IQR 5.1 to 20.6). Overall, 389 participants initiated on‐demand TDF/FTC PrEP regimen during the study; 199 participants initiated TDF/FTC during the blind phase while 161 participants from the placebo group and 29 new participants started TDF/FTC during the OLE phase. The characteristics of these participants are detailed in Table 1. All were born male with a median age of 35 years and 92% were white with a median BMI of 23 kg/m^2^. Only 4% had hypertension and 1% diabetes or dyslipidemia. The median follow‐up after TDF/FTC initiation was 19.2 months (IQR 18.0 to 26.9).

**Table 1:** Characteristics of the participants included in the study

Characteristics	Blind phase			All participants on TDF/FTC^a^ (N = 389)
	TDF/FTC (N = 199)	Placebo (N = 201)	*p* value	
Gender – no (%)				
Male	198 (99.5)	200 (99.5)		387 (99.5)
Transgender female	1 (0.5)	1 (0.5)		2 (0.5)
Median age (IQR) – year	35 (29 to 43)	34 (28 to 42)	0.56	35 (29 to 43)
Age group – no (%)				
18 to 29 years	57 (29)	57 (28)	0.88	105 (27)
30 to 39 years	72 (36)	73 (36)		142 (37)
40 to 49 years	50 (25)	55 (27)		103 (26)
>50 years	20 (10)	16 (8)		39 (10)
Site of enrolment – no (%)				
France			0.65	
Paris	96 (48)	105 (52)		189 (49)
Lyon	47 (24)	36 (18)		76 (20)
Nice	13 (7)	18 (9)		34 (9)
Tourcoing	13 (7)	14 (7)		28 (7)
Nantes	9 (5)	6 (3)		19 (5)
Montreal	21 (11)	22 (11)		43 (11)
Caucasian – no (%)	188 (94)	178 (89)	0.04	356 (92)
Use of recreational drugs^b^ – no (%)	86 (44)	92 (48)	0.51	160 (43)
≥5 Alcoholic drinks per day of consumption – no (%)	49 (25)	42 (21)	0.42	87 (23)
Medical history				
Diabetes – no (%)	1 (0.5)	2 (1)	1	3 (1)
Hypertension – no (%)	8 (4)	6 (3)	0.60	15 (4)
Dyslipidemia – no (%)	0 (0)	4 (2)	0.13	2 (1)
Concomitant drug use				
NSAID – no (%)	7 (3.5)	4 (2)	0.38	20 (5)
Antihypertensive – no (%)	8 (4)	6 (3)	0.60	14 (4)
Weight (kg) – median (IQR)	71 (65 to 80)	72 (65 to 80)	0.56	72 (66 to 80)
BMI (kg/m^2^) – median (IQR)	23 (21 to 25)	23 (21 to 25)	0.99	23 (21 to 25)
Creatinine (µmol/l) – median (IQR)	81 (75 to 88)	82 (73 to 87)	0.52	81 (74 to 88)
eGFR^c^ (mL/minute/1.73 m^2^) – median (IQR)	106 (97 to 115)	108 (96 to 115)	0.44	106 (97 to 115)
eGFR > 90 mL/min/1.73 m^2^ – no (%)	173 (87%)	169 (84%)	0.42	333 (86%)
eGFR < 90 mL/min/1.73 m^2^ – no (%)	26 (13%)	32 (16%)		56 (14%)

FTC, emtricitabine; IQR, interquartile range; NSAID, non‐steroidal anti‐inflammatory drugs; TDF, tenofovir disoproxil fumarate.

a All the participants who initiated TDF/FTC: 199 participants from the TDF/FTC arm, 161 from the placebo arm and 29 new participants included in the OLE phase

b recreational drugs that were reported in the past 12 months included ecstasy, crack cocaine, cocaine, crystal, speed, and γ‐hydroxybutyric acid or γ‐butyrolactone

c estimated glomerular filtration rate calculated by the Chronic Kidney Disease Epidemiology Collaboration equation.

### 3.2 Changes in eGFR in the blind phase of the IPERGAY study

**Figure 1:**
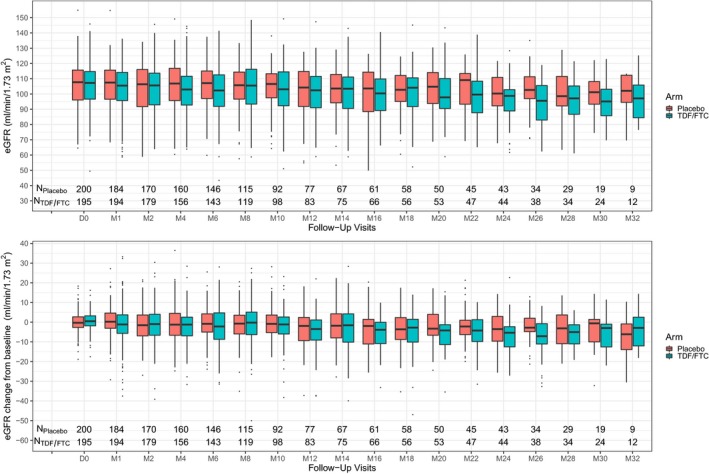
Variation over time of estimated glomerular filtration rate according to treatment arms in the blind phase of the IPERGAY trial. The figure depicts the change over time in eGFR estimated by CKD‐EPI equation (first panel) and the change of eGFR from baseline (second panel) by study months and treatment arm. Boxes encompass all data points between the 25th and 75th percentiles. Thick bars in boxes indicate the median data value. The upper whisker extends from the hinge to the largest value no further than 1.5 * Inter‐Quartile Range (IQR). The lower whisker extends from the hinge to the smallest value at most 1.5 * IQR of the hinge. Data beyond the end of the whiskers (‘outliers’) are plotted individually. The declining slope of eGFR from baseline was −0.88 mL/min/1.73 m^2^ per year in the placebo group (*p* = 0.04) and −1.53 mL/min/1.73 m^2^ per year in the TDF/FTC group (*p* < 0.01) and was no different between the two arms (*p* = 0.27). TDF denotes tenofovir disoproxil fumarate and FTC denotes emtricitabine.

**Figure 2:**
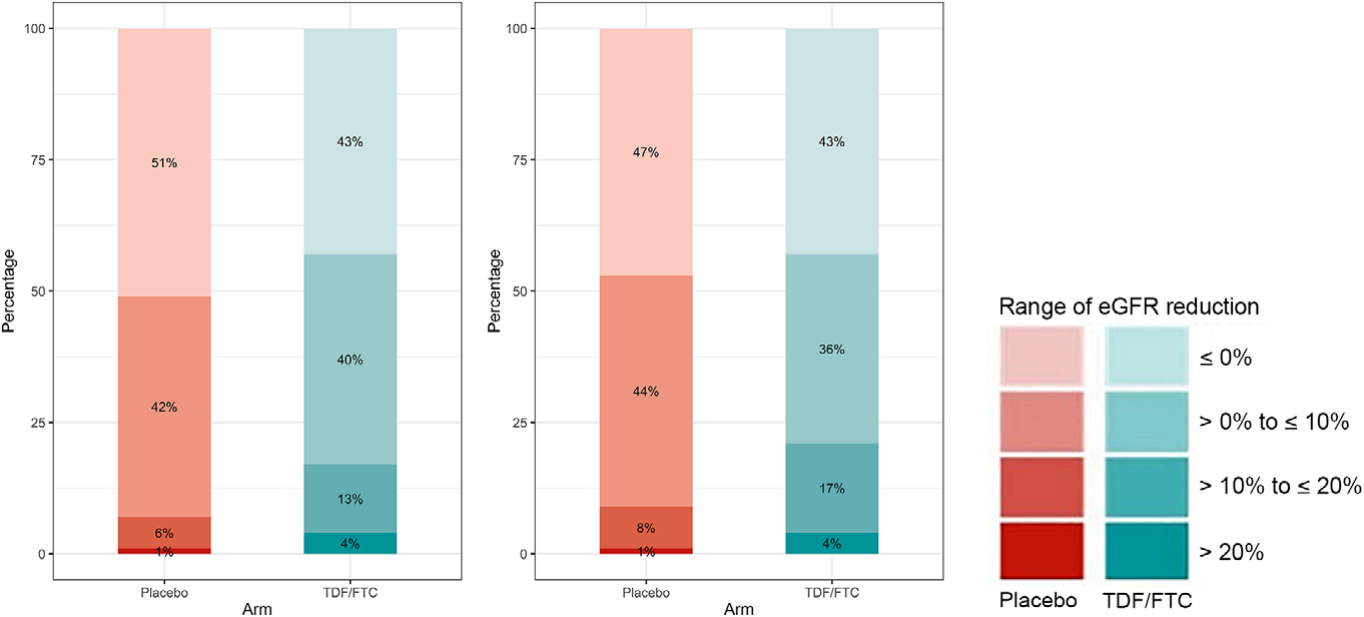
Distribution of estimated glomerular filtration rate changes from baseline at four weeks and six months according to treatment arms in the blind phase of the IPERGAY trial. The y‐axis and the percentages depicted inside the bars are the proportion in each treatment arm falling within the range of change in eGFR depicted to the right of the bars. TDF denotes tenofovir disoproxil fumarate and FTC denotes emtricitabine.

We observed a significant reduction in mean eGFR from baseline in the two arms with a declining slope of −0.88 mL/min/1.73 m^2^ per year in the placebo group (*p* = 0.04) and of −1.53 mL/min/1.73 m^2^ per year in the TDF/FTC group (*p* < 0.01). The slope difference between the two arms was 0.65 mL/min/1.73 m^2^ per year (*p* = 0.27). The changes over time in eGFR in both groups are represented in Figure 1. The distribution of eGFR changes at four weeks showed that most participants in both groups had a less than 10% decrease in eGFR (Figure 2). The proportion of participants with >10% eGFR decrease was 7% in the placebo group and 17% in the TDF/FTC group at four weeks (*p* < 0.01). These percentages remained quite similar at six months (placebo 9% and TDF/FTC 21%). Overall, among participants with baseline eGFR ≥ 70 mL/min/1.73 m^2^ (N = 190 and 195 with TDF/FTC and placebo respectively), 29 participants experienced an eGFR < 70 mL/min/1.73 m^2^ during the blind phase follow‐up; 9 in the placebo arm and 20 in the TDF/FTC arm (Logrank test *p* = 0.04) (Figure S1). None of the participants stopped treatment for renal adverse events during the blind phase.

### 3.3 Change in eGFR among participants starting TDF/FTC during both phases of the IPERGAY study (On TDF/FTC sample)

At baseline, the mean eGFR was 106 mL/min/1.73 m^2^ (IQR: 97 to 115). The mean slope of eGFR reduction from baseline was −1.14 mL/min/1.73 m^2^ per year (*p* < 0.001). At six months, 82% of the participants had a less than 10% decrease in eGFR, 14% had a decrease in eGFR of 10 to 20% and only 4% (n = 16) had a decrease of more than 20%. During follow‐up, an eGFR reduction to <70 mL/min/1.73 m^2^ occurred in 45 participants for a cumulative proportion of 14% (95% CI: 9 to 18%) by 24 months. Of these 45 participants, two had no further follow‐up and 43 had a median of further follow‐up of 16 months (IQR: 10 to 26). Of these 43 participants, 22 (51%) had just a single episode of eGFR reduction to <70 mL/min/1.73 m^2^ with no further recurrence and only 12 participants had two consecutive measurements of eGFR < 70 mL/min/1.73 m^2^. The risk of having a second measurement of eGFR below 70 mL/min/1.73 m^2^ within one year after the first episode was 46% (95% CI: 28 to 60%) (Figure S2). Using a threshold of 60 mL/min/1.73 m^2^, only 15 participants had an eGFR < 60 mL/min/1.73 m^2^ during the study, and among 13 participants with further follow‐up, 10 had only a single value below this threshold.

### 3.4 Association between baseline risk factors and eGFR decline

Baseline risk factors significantly associated with a lower eGFR at baseline were an age >40 years, and hypertension. However, these risk factors were not associated with a significantly greater eGFR reduction over time (Table 2). Only recreational drugs users tended to have a greater eGFR reduction (−1.55 vs. −0.88 mL/min/1.73 m^2^ per year, *p* = 0.10). This trend persisted in multivariate analysis including all risk factors associated either with baseline eGFR (*p* = 0.07) or with eGFR decline (*p* < 0.30).

**Table 2:** Baseline risk factors associated with eGFR reduction among all participants initiating on‐demand TDF/FTC based PrEP

	PY	Estimated Mean eGFR at baseline (mL/min/1.73 m^2^)	Univariate model				Multivariate model^a^	
			*p* value	Slope of eGFR reduction per year (mL/min/1.73 m^2^) (±SE)	Slope difference in eGFR per year (mL/min/1.73 m^2^) (±SE)	*p* value	Slope difference in eGFR per year (mL/min/1.73 m^2^) (±SE)	*p* value
Baseline eGFR								
eGFR > 90 mL/min/1.73 m^2^ (n = 333)	633	108	<0.001	−1.22 (±0.21)	0.66 (±0.57)	0.25	0.43 (±0.59)	0.47
eGFR ≤ 90 mL/min/1.73 m^2^ (n = 56)	104	84		−0.56 (±0.53)				
Baseline age								
>18 to ≤40 years (n = 247)\	448	110	<0.001	−1.35 (±0.25)	0.56 (±0.39)	0.16	0.55 (±0.41)	0.19
>40 years (n = 142)	290	94		−0.79 (±0.30)				
White race								
Yes (n = 356)	685	104	0.09	−1.15 (±0.20)	0.13 (±0.80)	0.88		
No (n = 33)	53	108		−1.02 (±0.78)				
Hypertension								
No (n = 347)	709	105		−1.19 (±0.20)	1.29 (±0.99)	0.20	1.52 (±1.00)	0.14
Yes (n = 15)	28	93		0.10 (±0.97)				
Baseline body mass index								
18 to 25 kg/m^2^ (n = 282)	534	104		−1.13 (±0.23)		0.65^b^		
<18 kg/m^2^ (n = 12)	18	124		−2.45 (±1.41)	−1.32 (±1.42)			
>25 kg/m^2^ (n = 93)	183	103	<0.001	−1.09 (±0.39)	0.04 (±0.45)			
Missing (n = 2)	3							
Use of recreational drugs								
No (n = 215)	417	104	0.26	−0.88 (±0.25)	−0.67 (±0.40)	0.10	−0.76 (±0.40)	0.07
Yes (n = 160)	301	105		−1.55 (±0.30)				
Missing (n = 14)	21							
≥5 Alcoholic drinks per day of consumption								
No (n = 294)	562	104	0.25	−1.31 (±0.22)	0.68 (±0.46)	0.15	0.91 (±0.47)	0.06
Yes (n = 87)	166	106		−0.63 (±0.41)				
Missing (n = 8)	10							

ASE standard error; PY persons‐years; eGFR estimated glomerular filtration.zrate; n number of participants.

a Multivariate analysis included baseline eGFR × time, baseline age × time, race, hypertension × time, BMI, use of recreational drugs at baseline × time, alcoholic drinks × time.

### 3.5 Relationship between recent tenofovir exposure and eGFR

A higher exposure to TDF/FTC prior to the visit was associated with a lower eGFR at the time of the visit (Table 3). Participants who had taken >15 pills per month in the prior two months had a lower eGFR at the following visit when compared to those who had taken ≤15 pills (eGFR difference: −0.88 mL/min/1.73 m^2^, *p* < 0.001). Similarly, higher tenofovir plasma concentrations, which reflect TDF/FTC exposure in the prior week, were associated with lower eGFR at the same visit following a dose‐response relationship (eGFR difference compared to tenofovir level <2 ng/mL: −0.98, −1.28, and −1.82 mL/min/1.73 m^2^, for >2 to ≤10 ng/mL, >10 to ≤40 ng/mL and >40ng/mL respectively, *p* < 0.001). This association persisted when the analysis was adjusted for time, age, eGFR and hypertension at baseline.

**Table 3:** Relationship between recent tenofovir exposure and eGFR at the following visit among all participants initiating on‐demand TDF/FTC based PrEP

	PY	Univariate model^a^		Adjusted analysis^b^	
		Estimation of the effect on eGFR (mL/min/1.73 m^2^) (±SE)	*p* value	Estimation of the effect on eGFR (mL/min/1.73 m^2^) (±SE)	*p* value
Number of pills per months in the last two months^c^					
≤ 15 pills (n^d^ = 1941)	255	Reference			
> 15 pills (n^d^ = 2279)	370	−1.38 (±0.30)	<0.001	−0.88 (±0.30)	<0.01
Missing	112				
Tenofovir plasma concentration^c^					
≤ 2 ng/mL (n^d^ = 1714)	321	Reference			
> 2 to ≤10 ng/mL (n^d^ = 327)	50	−1.27 (±0.50)	<0.001^e^	−0.98 (±0.49)	<0.001^e^
> 10 to ≤40 ng/mL (n^d^ = 512)	80	−1.42 (±0.42)		−1.28 (±0.42)	
> 40 ng/mL (n^d^ = 2231)	351	−2.06 (±0.30)		−1.82 (±0.30)	
Missing	25				

eGFR, estimated glomerular filtration rate; n, number of visit; PY persons‐years; SE standard error.

a For univariate analysis, we used a linear mixed effects model

b linear mixed model adjusted for time, age >40 years, baseline eGFR < 90 mL/min/1.73 m^2^ and hypertension. Interactions terms between age or baseline eGFR and tenofovir exposure were not statistically significant and were thus not included in the final model.

c time‐dependent variables

d number of visits

e global *p* value.

### 3.6 Association between the amount of pills used and eGFR decline

During the study, 102 participants who had taken 15 pills or less per month at ≥75% of visits were considered “low PrEP users.” Analysis adjusted for age showed that eGFR decline was −0.55 and −1.25 mL/min/1.73 m^2^ per year in low users versus other participants respectively (*p* = 0.16).

### 3.7 Serious renal adverse events and urine biomarkers of renal dysfunction on TDF/FTC

**Figure 3:**
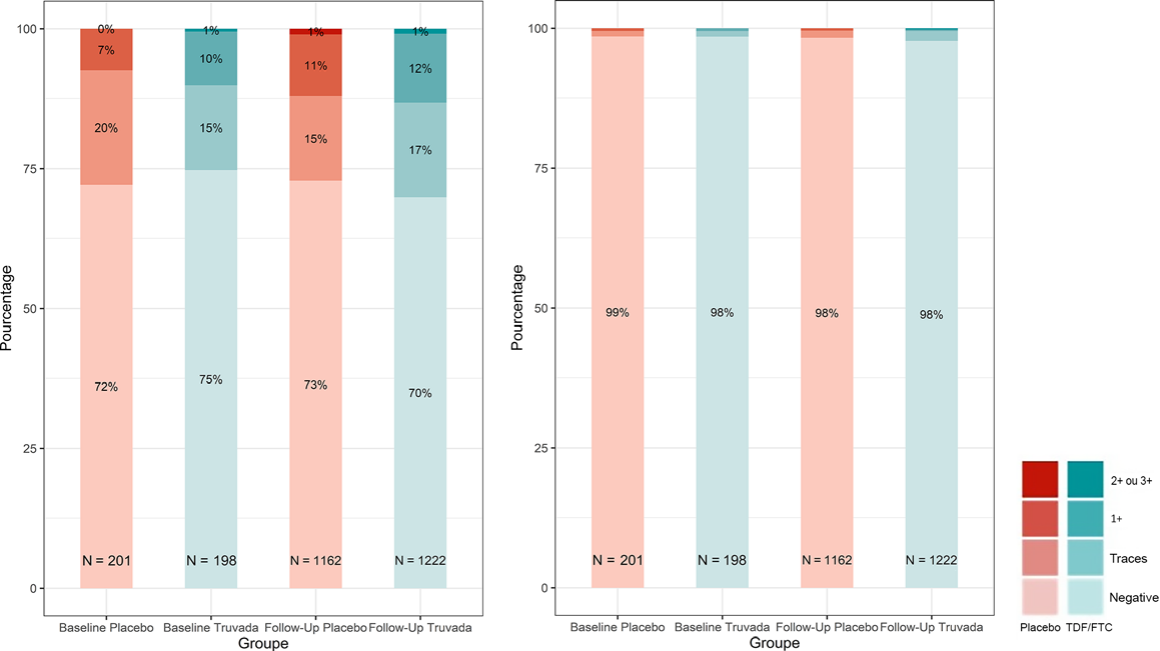
Markers of proximal tubulopathy according to treatment arms in the blind phase of the IPERGAY trial. ^a^Median of follow‐up of 9.4 months (IQR 5.1 to 20.6). Proportion of study visits with protein and glucose in urine dipsticks at baseline and during the follow‐up (median of 9.4 months) in the placebo and TDF/FTC arm. Numbers inside the bars are the number of urine dipstick accumulated after starting treatment.

**Figure 4:**
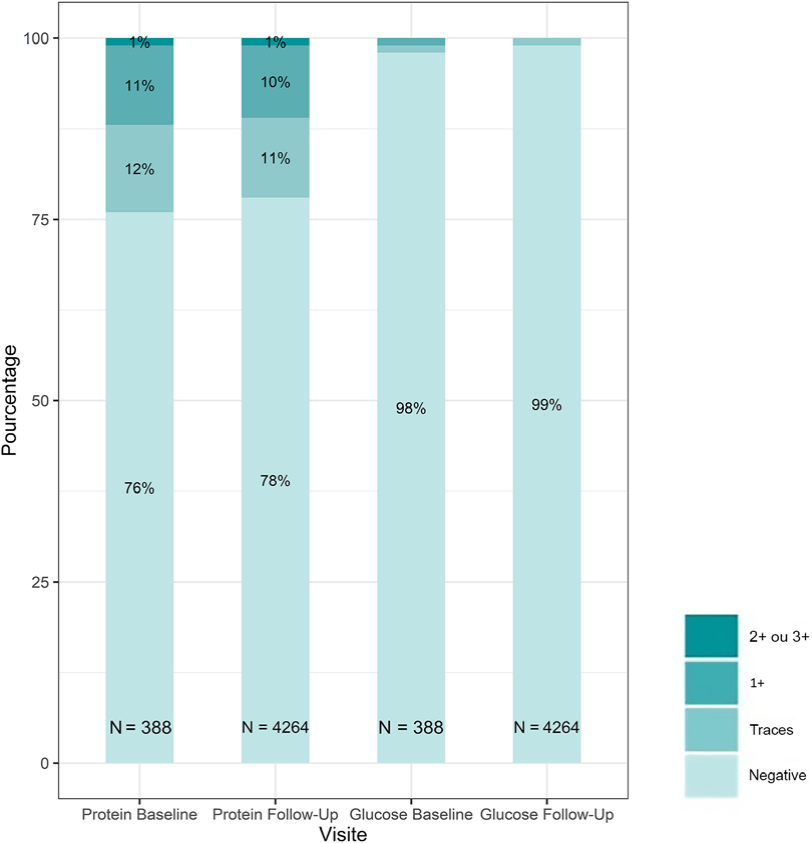
Markers of proximal tubulopathy among all participants initiating on‐demand TDF/FTC based PrEP. ^a^Median of follow‐up of 19·2 months (IQR 18·0 to 26·9). Proportion of study visits with protein and glucose in urine dipsticks at baseline and during the follow‐up (median of 19·2 months) in all participants initiating TDF/FTC. Numbers inside the bars are the number of urine dipsticks accumulated after starting TDF/FTC.

A creatinine elevation of grade 1 occurred in 72 participants (19%) and two participants (1%) have had a grade 2 elevation (Table 4). On the five participants who discontinued TDF/FTC during the study, three participants (<1%) had an event related to kidney dysfunction: two participants had a grade 1 kidney adverse events (with a decline in eGFR to 58 mL/min/1.73 m^2^ and 49 mL/min/1.73 m^2^) and one participant had a grade 2 (with a decline to 39 mL/min/1.73 m^2^). In the blind phase, 1222 urine dipsticks were collected in the TDF/FTC group and 1162 in the placebo group. During follow‐up, the proportion of dipstick proteinuria ≥2+ was similar in the two groups (placebo 1%, TDF/FTC 0.9%), as well as the proportion of dipsticks with glycosuria ≥2+ (placebo <1%, TDF/FTC <1%) (Figure 3). The proportion of dipstick proteinuria or glycosuria ≥2+ was also low in the 4264 dipsticks done after participants started TDF/FTC (Figure 4). One participant had proteinuria and glycosuria ≥2+ during the study but not on the same visit. None of the participants discontinued TDF/FTC for tubulopathy and no case of Fanconi syndrome was reported.

**Table 4:** Kidney adverse events during the blind phase and in all participants initiating TDF/FTC in the IPERGAY study

	Blind phase			All participants on TDF/FTC^a^ (N = 389)
	Placebo (n = 201)	TDF/FTC (n = 199)	*p* value	
Median follow‐up – months (IQR)	9.0 (5.4 to 20.3)	9.8 (4.7 to 21.1)		19.2 (18 to 26.9)
Any adverse event no – %	182 (91%)	188 (94%)	0.14	380 (98%)
Any serious adverse event no %	17 (8%)	21 (11%)	0.48	58 (15%)
Treatment discontinuation due to adverse event no – %	0	1 (<1%)	0.50	5 (1.3%)
Treatment discontinuation for kidney adverse event no – %	0	0		3^b^ (<1%)
Creatinine elevation no – %	20 (10%)	35 (18%)	0.03	74 (19%)
Grade 1	19 (9%)	35 (18%)		72 (19%)
Grade 2	1 (<1%)	0		2 (<1%)
Grade 3	0	0		0
Grade 4	0	0		0
Tubulopathy markers no – %				
Proteinuria ≥2+	9 (4%)	11 (6%)	0.66	39 (10%)
Glycosuria ≥2+	0	1 (<1%)	0.50	3 (<1%)

FTC, emtricitabine; TDF, tenofovir disoproxil fumarate.

a All the participants who initiated TDF/FTC: 199 participants from the TDF/FTC arm, 161 from the placebo arm and 29 new participants including in the OLE phase

b two participants had a grade 1 kidney adverse event (with a decline in eGFR to 58 mL/min/1.73 m^2^ and 49 mL/min/1.73 m^2^) and one participant had a grade 2 event (with a decline to 39 mL/min/1.73 m^2^).

## 4 Discussion

In our study, the renal safety of on‐demand PrEP with TDF/FTC among young MSM was very good. Indeed, after a median follow‐up of 19.2 months on TDF/FTC, less than 1% of participants (n = 3) discontinued TDF/FTC because of a decline in eGFR < 60 mL/min/1.73 m^2^. On‐demand PrEP was not associated with significant emerging proteinuria or glycosuria and no Fanconi syndrome was reported. The proportion of participants with dipstick proteinuria or glycosuria ≥2+ was 1% at baseline and did not increase on TDF/FTC which is consistent with previously reported studies with daily PrEP 10, 24, 25.

During the blinded phase of our study, eGFR decline from baseline was not significantly different between the TDF/FTC and placebo groups (−0.88 and −1.53 mL/min/1.73 m^2^ per year respectively, with a slope difference of only 0.65 mL/min/1.73 m^2^ per year (*p* = 0.27). In addition, the mean slope of eGFR reduction from baseline was only −1.14 mL/min/1.73 m^2^ per year when considering all 389 participants who started TDF/FTC during both study phases, and was not significantly different according to age or eGFR at baseline. This small reduction in eGFR in our study with on‐demand TDF/FTC seems to be lower than those reported with daily PrEP at one year: −2.09 mL/min/1.73 m^2^ in the iPrEx trial among participants with high adherence 10, −2.01 mL/min/1.73 m^2^ in the Partners PrEP trial 12 and −1.70 mL/min/1.73 m^2^ in the Bangkok Tenofovir Study 11. In the Discover trial, the mean reduction in creatinine clearance at one year in the TDF/FTC arm was −2.30 mL/min 26. The mean eGFR reduction was even more pronounced in the EPIC – NSW study (−4.5 mL/min/1.73 m^2^ per year) 27.

The overall reduction and intermittent exposure to TDF/FTC in our study as compared to a daily regimen, may explain the better renal safety of on‐demand PrEP. As already reported with daily PrEP 13, 15, we found a dose‐response relationship between recent TDF exposure (prior two months or prior week) and a lower eGFR at the following visit, which strongly supports that an overall reduction of exposure to TDF/FTC may have a positive impact on renal function. The intermittent nature of TDF/FTC exposure is also important to consider with on‐demand PrEP according to the reported large inter and intra‐participant variability in pill use over time in the study 6. On‐demand regimen may promote short periods with no exposure to TDF/FTC allowing reversibility of glomerular renal function decline before a new exposition, including in users with a high level of exposure to TDF/FTC. Indeed, partial reversibility of renal dysfunction upon TDF discontinuation has already been reported in patients with HIV‐infection 18 and among daily PrEP users. Mugwanya et al. showed that 70% and 96% of participants in the Partners PrEP study had a confirmed >75% of eGFR rebound to baseline level by four and eight weeks respectively after drug discontinuation 19. This phenomenon may explain why no significant difference in eGFR decline was seen in our study between “low PrEP users” and other participants. It is also important to underline that the intermittent exposure to TDF/FTC with on‐demand PrEP was associated with a strong reduction in the relative risk of HIV infection, up to 97% with a median number of 18 pills per month 20. These data suggest that on‐demand PrEP may maximize the renal safety of TDF while providing a high level of protection against HIV.

Although daily PrEP with TDF/FTC is associated with a slight decline in eGFR, meta‐analysis of clinical trials showed that the use of TDF/FTC in PrEP remains safe 28. Most of the participants included in these clinical trials were young with normal renal function at baseline and no comorbidities. Compared to daily PrEP, the benefit of on‐demand regimen in terms of preservation of renal function is likely to be negligible in these users. However, with the global roll‐out of PrEP programmes, new PrEP users have emerged with higher risk of kidney dysfunction on TDF. Previous studies on daily PrEP highlighted that a baseline age >40 years or a baseline eGFR ≤ 90 mL/min/1.73 m^2^ were associated with a greater eGFR reduction 13, 14, 15. In our study, we did not find that participants at‐risk of renal dysfunction (age >40 years, eGFR at baseline <90 mL/min/1.73 m^2^ or hypertension) had a significantly greater slope of eGFR reduction over time, suggesting that on‐demand PrEP may remain safe in this population. In the DISCOVER trial, daily PrEP with tenofovir alafenamide (TAF)/FTC was as effective as TDF to prevent HIV acquisition but was not associated with any creatinine clearance reduction at one year 26. These data suggest that TAF/FTC will be probably the safest option for PrEP users with markers of renal vulnerability. However, its cost and availability may be important barriers for its use and on‐demand PrEP with TDF/FTC may remain a valuable alternative.

Our study has several limitations. First, participants were not randomized in sub‐groups by TDF exposure, which makes more difficult to model, retrospectively, the interaction between TDF exposure and eGFR decline, especially as TDF exposure was highly variable for each participant during follow‐up due to the on demand dosing regimen which was linked to sexual activity. Second, participants included in the IPERGAY trial were young, caucasian, with few comorbidities and had normal renal function at baseline. Other studies are required to precise the safety of on demand PrEP with TDF/FTC in participants with altered renal function, other ethnicity, using nephrotoxic medications or with comorbidities such as diabetes or hypertension. Third, the proportion of TDF‐induced tubulopathy may have been underestimated in our study by the lack of sensitivity of urine dipstick to detect proximal tubular dysfunction. A recent study conducted in participants of the IPrEx cohort showed that daily‐PrEP may be associated with markers of renal tubular dysfunction y^25^. The long term impact of such change is unknown and additional data are needed in the setting of on‐demand PrEP. Fourth, the median follow‐up in our study was only 19 months which could be too short to assess the long term renal impact of on‐demand PrEP since PrEP use might last for several years in people at risk of HIV acquisition. Finally, we did not perform a direct measurement of GFR in our study, which is a common limitation in a large cohort of participants. The study would have gained to assess eGFR decline by a direct measure of GFR in a sample of the population.

## 5 Conclusions

In the setting of the ANRS‐IPERGAY PrEP trial conducted among MSM in France and Canada, the renal safety of on‐demand PrEP was very good. Relative to daily PrEP, the overall reduction and intermittent exposure to TDF/FTC with such regimen may explain this beneficial outcome and have clinical relevance on the long term.

## Competing interests

JMM has participated to advisory boards for Gilead, Merck, ViiV and Teva and his institution has received research grants from Gilead. GP has received consulting fees from Gilead, Boehrringer Ingelheim, Nephrotek, ViiVhealtcare, Abbvie, MSD and Bristol Myers Squibb. LC has received personal fees and non‐financial support from Gilead Sciences, Janssen Cilag, Abbvie, MSD, ViiV Healthcare outside the submitted work. CT reports receiving support from Gilead Sciences and Pfizer. EC reports receiving support as an adviser for Janssen, MSD, and ViiV Healthcare, and non‐financial support from Gilead Sciences. FR has received personal fees from Gilead, Janssen, Merck, Mylan and ViiV Healthcare, outside the submitted work. All other authors declare no competing interests.

## Author's contributions

J‐MM and GL designed the study. GL wrote the first draft of the report. GA, LM and IC designed the analysis. J‐MM, GL, GA, LM, IC and CCa analysed the data. CCa coordinated data management. J‐MM, GP, CC, CT, EC, ES, LC, FR took part in the study at their sites. All authors critically reviewed and approved the manuscript.

## Supporting information


**Figure S1.** Cumulative probability to have one measurement of estimated glomerular filtration rate falling below 70 mL/min/1.73 m² according to the treatment arm during the blind phase of the IPERGAY trial.
**Figure S2.** Cumulative probability to have one measurement of estimated glomerular filtration rate falling below 70 mL/min/1.73 m² among all participants initiating on‐demand TDF/FTC based PrEP during the IPERGAY trial.
