# Supplementary material for: Changes in kidney function among men having sex with men starting on demand tenofovir disoproxil fumarate – emtricitabine for HIV pre‐exposure prophylaxis
Source: J Int AIDS Soc. 2020 Feb 22;23(2):e25420. doi: 10.1002/jia2.25420 (PMC7035456; doi:10.1002/jia2.25420)
Supplement: Supplementary file 1 — Figure S1. Cumulative probability to have one measurement of estimated glomerular filtration rate falling below 70 mL/min/1.73 m² according to the treatment arm during the blind phase of the IPERGAY trial. Figure S2. Cumulative probability to have one measurement of estimated glomerular filtration rate falling below 70 mL/min/1.73 m² among all participants initiating on‐demand TDF/FTC based PrEP during the IPERGAY trial. [file JIA2-23-e25420-s001.docx]

**Appendix**

**Figure S1. Cumulative probability to have one measurement of estimated glomerular filtration rate falling below 70mL/min/1.73m²** according to the treatment arm during the blind phase of the IPERGAY trial.

**Figure S2. Cumulative probability to have one measurement of estimated glomerular filtration rate falling below 70mL/min/1.73m²** among all participants initiating on-demand TDF/FTC based PrEP during the IPERGAY trial.
